# Gayet-Wernicke Encephalopathy: An Unusual Complication of Vomiting

**DOI:** 10.7759/cureus.17604

**Published:** 2021-08-31

**Authors:** Hanane Delsa, Amine Benfaida, Zakaria Salimi, Fedoua Rouibaa, Jehanne Aasfara

**Affiliations:** 1 Gastroenterology and Hepatology, Cheikh Khalifa International University Hospital, Mohammed VI University of Health Sciences (UM6SS), Casablanca, MAR; 2 Neurology, Cheikh Khalifa International University Hospital, Mohammed VI University of Health Sciences (UM6SS), Casablanca, MAR

**Keywords:** gayet-wernicke encephalopathy, vomiting, thiamine, magnetic resonance imaging, vitamin treatment

## Abstract

Gayet-Wernicke encephalopathy (WE) is a serious and acute disease of the central nervous system caused by thiamine (vitamin B1) deficiency. Multiple etiologies are indicated, although alcohol abuse is the most reported cause. If not treated promptly, WE can lead to serious complications such as Korsakoff's syndrome, coma, or death. This diagnosis should be considered even without a history of alcohol dependence. We describe two cases of non-alcohol related WE complicating vomiting caused by different etiologies. The diagnosis was suspected on clinical presentation and confirmed by brain MRI and effective response to parenteral administration of thiamine.

## Introduction

Gayet-Wernicke encephalopathy (WE) is a severe neurological complication caused by thiamine deficiency, which can be life-threatening. Several causes have been described, the most common of which is chronic alcoholism. However, many situations can cause this disease like fasting, starvation, and malnutrition [1]. WE can lead to serious complications such as Korsakoff's syndrome, coma, or even death. To avoid these devastating outcomes, the diagnosis must be made early even in non-alcoholic patients, especially those with digestive symptoms [2].

We describe two cases of non-alcoholic WE discovered in the context of severe vomiting. The first patient was a young woman with hyperemesis gravidarum, and the second patient presented with a malignant gastro-intestinal obstruction with severe vomiting. A diagnosis of WE was made based on clinical presentation, brain MRI, and decreased plasma thiamine levels. Medical treatment was introduced early with good outcomes.

## Case presentation

Case 1

**Figure 1:**
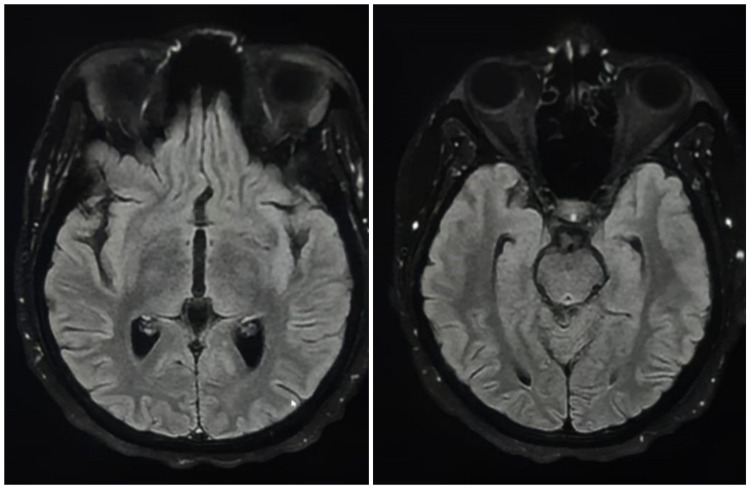
Axial sections of brain MRI in fluid-attenuated inversion recovery (FLAIR) sequence: normal

A 21-year-old woman at 15 weeks of pregnancy, with no history of alcohol consumption, was admitted to the emergency room for persistent vomiting evolving over a two-week period. The clinical status of the patient worsened gradually; she presented ataxia, horizontal nystagmus, dizziness, confusion, and bradypsychia. A brain MRI was performed which was normal (Figure 1).

The diagnosis of hyperemesis gravidarum complicated with a WE was made, based on clinical presentation and good outcome after treatment. The blood test showed a low thiamine level. The patient was treated with high-dose intravenous thiamine (500 mg every eight hours for three days, afterwards, 250 mg every eight hours for five days and transitioned to oral supplementation) and symptomatic treatment (antiemetic drugs and infusions containing dextrose was avoided).

The patient’s condition improved with a complete recovery from confusion and ataxia.

Case 2

A 60-year-old man with a chronic smoking history was admitted to the emergency room for an acute confusional state evolving over two days in the context of severe vomiting.

**Figure 2:**
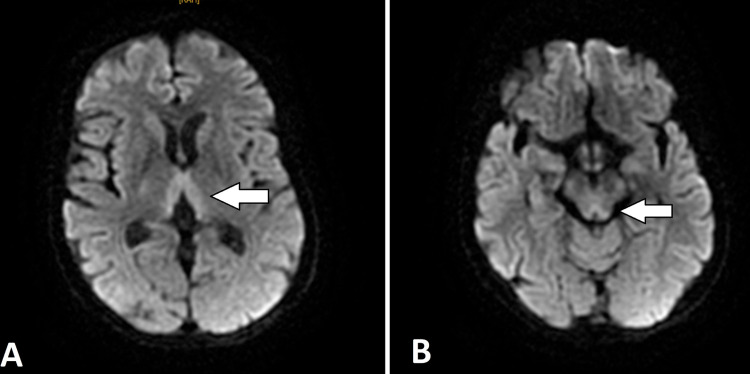
Axial sections of brain MRI in diffusion sequence B1000 Hyper signal abnormalities on medial thalamus (A) and the mammillary bodies (B).

**Figure 3:**
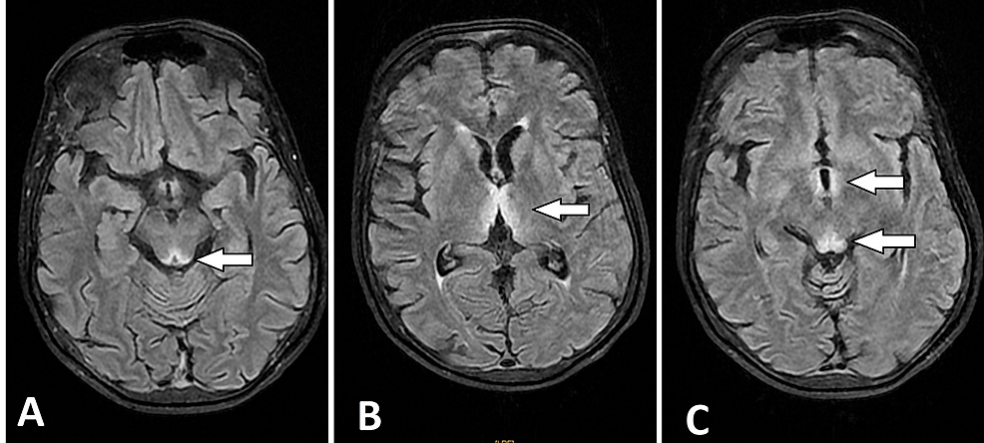
Axial sections of brain MRI In fluid-attenuated inversion recovery (FLAIR) sequence Symmetrical high-signal intensity of the tectum of the midbrain, the periaqueductal gray matter (A), the medial thalami (B) and periventricular region of the third ventricle (C)

Clinical examination revealed ataxia, ophthalmoplegia, nystagmus, and dysarthria. A brain MRI was performed and revealed symmetrically hyperintensity around the third ventricle and in the mammillary bodies and periaqueductal grey matter (Figures 2-3).

Full laboratory assessment revealed ferriprive anemia, hypokalemia, and normal serum levels of B1 vitamin, folic acid, and B12 vitamin.

**Figure 4:**
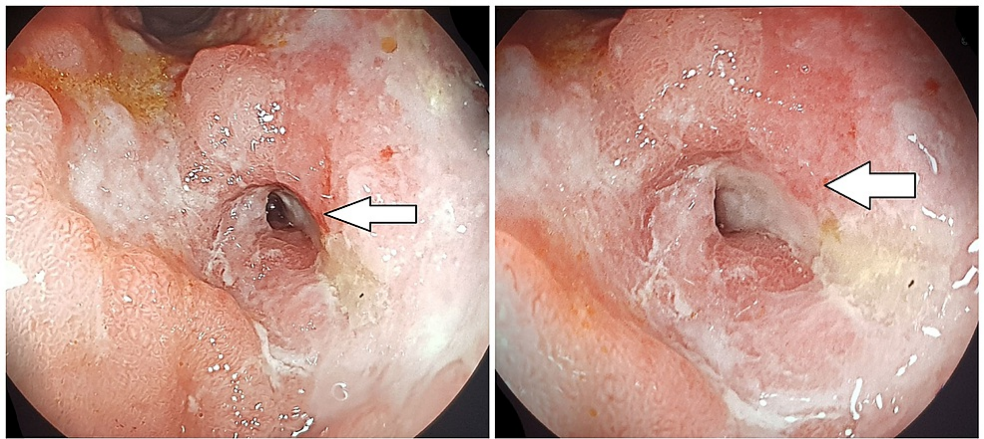
Endoscopic view of gastric carcinoma with pyloric stenosis (white arrow)

This clinical presentation was attributed to WE based on typical neurological symptoms and radiological findings. Vitamin intravenous thiamin treatment and nutritional supplementation were initiated with good clinical outcomes. The neurological signs resolved completely and the patient was entirely independent without sequelae. Esophagogastroduodenoscopy found a malignant mass of antrum with pyloric stenosis (Figure 4). Biopsy confirmed the presence of gastric adenocarcinoma.

The staging showed no metastasis. The patient underwent a gastrectomy without postoperative complications.

## Discussion

WE was first described in 1881 by Carl Wernicke as a clinical triad: confusional state, oculomotor disorders (ophthalmoplegia and nystagmus), and cerebellar ataxia. However, this triad is only complete in 8%-30% of cases [3], making the diagnosis of WE difficult. Ocular abnormality is a sign suggesting WE but occurs in only 15%-29% of patients [4,5]. Nystagmus or ophthalmoplegia are very common signs [5]. Ataxia which usually presents as a gait disturbance is observed in 23%-25% of patients [5]. This serious and acute central nervous system disease is caused by a deficiency of thiamine (vitamin B1) which can result from several etiologies. The most common cause of WE is alcohol abuse but the diagnosis must be evoked in all patients with severe vomiting essentially hyperemesis gravidarum, renal disease, visceral surgery mainly bariatric, and all pathologies causing gastro-intestinal obstruction [6,7].

In patients with nutritional deficiency and non-alcohol abuse history, clinicians must be aware of the high risk of WE [7]. Vitamin B1 is involved in the Krebs cycle which provides most of the energy needs of the cell. Thiamine triphosphate would act as a neurotransmitter of nerve impulses, which would explain the neurological disorders of vitamin B1 deficiencies by the alteration of the blood-brain barrier and the production of anti-free radicals responsible for cell death by necrosis and apoptosis [4,5]. We could, by definition, expect a low level of thiamine in WE. Nevertheless, thiamine tests have well-known limits. Serum thiamine levels are a bad measure of thiamine status [8]. They do not necessarily reflect intracerebral thiamine levels [8]. In our patients, only one had a low level of thiamine. In these particular cases, an MRI must be performed early in front of any clinical suspicion of WE.

In brain MRI, abnormalities due to thiamine deficiency are objectified by bilateral and symmetrical lesions and manifested by hyper signal intensities on the T2 and fluid-attenuated inversion recovery (FLAIR) sequences. The specific locations are the paraventricular region of the thalamus, the hypothalamus, the mammillary bodies, the peri-aqueductal region, around the third ventricle and, cerebellar vermis [9,10]. However, brain MRI has a sensitivity of 53%, but higher specificity of 93% [9]; a normal imaging should not exclude the diagnosis. In light of these highly suggestive radiological signs, the initiation of thiamine therapy should not be delayed in the WE to improve the prognosis.

Studies by Thompson and Cook support several different regimens for patients with WE and those at risk of developing it [11,12]. They should be treated empirically with a minimum of 500 mg thiamine hydrochloride (dissolved in 100 ml of normal saline). This treatment should be given by infusion over a period of 30 min, three times per day for 2-3 days. If there is no response, supplementation may be discontinued after 2-3 days. Nevertheless, if an effective response is observed, 250 mg thiamine given intravenously or intramuscularly daily for 3-5 days, or until clinical improvement, should be continued. Associated magnesium supplementation is recommended, as it is a cofactor of vitamin B1 and magnesium deficiency is a cause of resistance to treatment [5,13]. In our cases, both of our patients received this supplementation directly after the MRI, with good outcomes and complete resolution of symptoms.

## Conclusions

WE is a neurological complication of thiamine deficiency, especially in alcoholic patients. However, WE must be evoked in front of neurological symptoms like confusion, ataxia, or oculomotor signs, even in non-alcoholic patients presenting with gastrointestinal signs like vomiting. This unknown and severe complication was suspected in our patients and confirmed based on clinical presentation, brain MRI, and blood B1 vitamin level. The early treatment with high-dose thiamine allowed for the avoidance of irreversible complications and improved prognosis in our patients.
